# Fluoridated milk is effective in prevention of enamel caries in adolescents: a randomised trial

**DOI:** 10.2340/aos.v84.45271

**Published:** 2025-12-29

**Authors:** Madeleine Rohlin, Jessica Neilands, Julia R. Davies, Per-Erik Isberg, Claes Wickström, Gunnel Svensäter

**Affiliations:** a Department of Oral Biology, Faculty of Odontology, Malmö University, Malmö, Sweden; b Department of Statistics, Lund University, Lund, Sweden

**Keywords:** dental caries, radiography, bitewing, disease progression, classification, fluoridation, follow-up studies

## Abstract

**Objective:**

To investigate whether low daily doses of fluoridated milk, as an adjunct to oral hygiene routines with fluoridated toothpaste, can prevent caries development in enamel and dentine in adolescents.

**Material and methods:**

Adolescents (mean age 13 years) at three dental clinics in Sweden were enrolled to a randomised clinical trial (RCT) including baseline and 2-year follow-up examinations. The intervention group consumed milk supplemented with fluoride (0.75–1.0 mg) daily, while the control group consumed milk with water. Caries lesion development was assessed visually in line with the international caries detection and assessment system (ICDAS), except for proximal surfaces of premolars and molars which were assessed using a radiological classification system. Outcome measures were decayed, missing and filled surfaces (DMFS) increment, caries lesion arrest and progression.

**Results:**

Eighty seven participants in the intervention group and 72 in the control group completed the study. The intervention reduced the incidence of DMFS increment for enamel lesions, but not for dentine lesions. The degree of caries lesion arrest in outer enamel was higher in the intervention group compared to the control group, whereas caries lesion progression was lower in the intervention group.

**Conclusion:**

Fluoride exerts an effect on caries lesions in the outer enamel, and fluoridated milk thus can be beneficial to adolescents with such lesions.

**Trial registration:**

Clinicaltrials.gov registration number NCT06684405.

## Introduction

The burden of dental caries remains a major health challenge with over 35% of the global population affected by the presence of cavities in permanent teeth [1]. A meta-analysis of studies of caries status in databases from 2000 through 2021 concluded that 77% of European adolescents were affected by caries with a significantly higher prevalence in the 16–19-year-old group [2]. At the individual level, there was a heterogeneity in experience of enamel caries towards few individuals with a high number of lesions [2].

There is convincing evidence that repeated topical application of fluoride plays an important role in caries prevention [3, 4]. When fluoride is adsorbed to the hydroxyapatite crystal surface, it will become more resistant to low pH generated by acid production in dental biofilms. Furthermore, fluoride adsorbed to the crystal surface will attract calcium ions and thereby promote enamel remineralisation [5, 6]. Topical fluoride can also provide an antimicrobial action. From early laboratory studies, it was concluded that fluoride effectively inhibits sugar transport and glycolysis thereby reducing acid production in oral streptococci [7, 8]. In addition, diffusion of hydrogen fluoride into the cytoplasm in combination with inhibition of proton-extruding adenosine triphosphate (ATP)ases, accelerates internal acidification of the cytoplasm further inhibiting metabolic acid production [9]. Recent *in vitro* studies show that exposure of multi-species biofilms derived from saliva to 500 ppm fluoride rapidly inhibits the drop in the pH seen in response to sucrose, confirming that fluoride interferes with bacterial acid production and thereby the driver of caries development [10]. This finding suggests that the antimicrobial effect of fluoride on dental biofilms contributes to caries prevention.

Milk fluoridation offers benefits in specific demographic groups and in countries where water fluoridation is not implemented. The bioavailability of added fluoride in different types of milk has been investigated in an amount of non-clinical published research and has been shown to be satisfactory [11]. A systematic review of clinical studies by Cagetti et al. [12] reported caries-preventive effect of milk fluoridation in pre-school children from two studies with medium grading [13, 14]. However, another review including 18 studies of children found that the reduction in dentine caries incidence varied from no [15, 16] to 70–90% effect [17]. A Cochrane review of fluoridated milk published in 2015 [6] included only one randomised study [18] which was carried out on children aged 3 years. As the study was published as an abstract only and the outcome data were incomplete, it was concluded that the study presented a high risk of bias.

From our search of the scientific literature, it is evident that there is no report of caries prevention with fluoridated milk in adolescents. As milk represents an important part of adolescents’ diet in many countries, the use of milk as a vehicle has some attraction. In Sweden where fluoride is not added to the public water supply and most adolescents drink cold milk daily with their meals, fluoridated milk may constitute one alternative in caries prevention to a targeted group of adolescents. The objective of the present study was to investigate whether low doses of fluoride administered daily in milk, in addition to oral hygiene routines with fluoride toothpaste, can have preventive effect on caries development in adolescents.

## Methods

### Design

**Figure 1:**
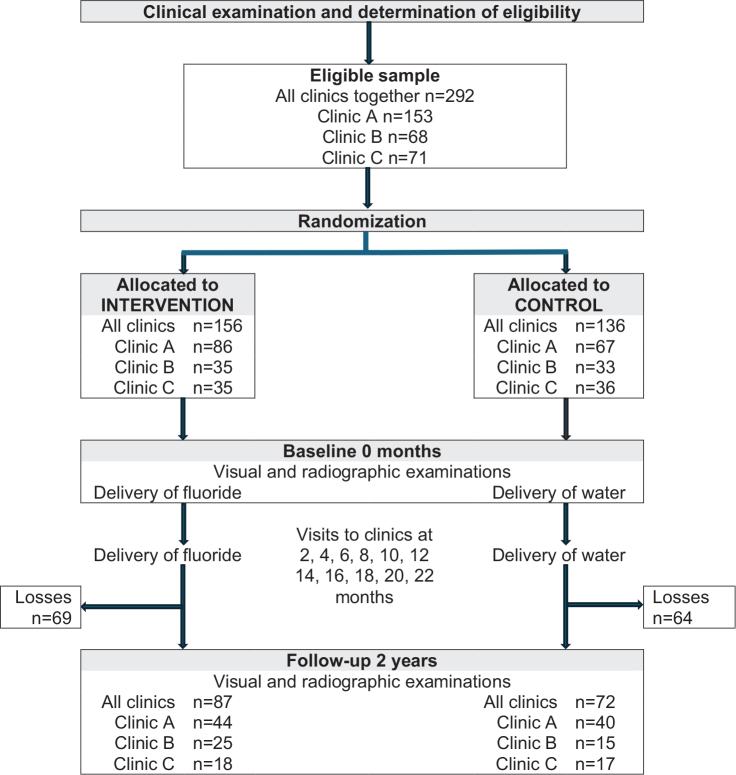
Study design.

This was a 2-year randomised controlled trial (RCT) in line with the Consolidated Standards of Reporting Trials (CONSORT) guidelines [19]. Figure 1 presents the study design with two groups; an intervention group and a control group in a 1:1 ratio. The unit of equal randomisation was the adolescent subject. The subjects of the intervention group received a fluoride water-solution to be added to milk and the subjects of the control group received water to be added to milk.

### Participants

Adolescents fulfilling the following criteria were included:

- subjects of both sexes, aged 12–13 years.
- no systemic disease.
- permanent posterior teeth and second premolars in contact with the mesial surface of first molars.
- adolescents who, together with active parents or guardians, gave informed written consent to participate. Written information on data confidentiality and the right to withdraw from the study at any time were given to all participants and their parents or guardians.

Adolescents were recruited during their scheduled annual appointment at one of three public dental clinics located in rural communities in three healthcare regions in southern Sweden.

### Intervention

The subjects in the intervention group received tubes with daily doses of sodium fluoride solution (0.75 mg for subjects aged 12 years and 1.0 mg for subjects when they were or turning 13 years according to recommendations by the Swedish Public Dental Health Agency) in 0.5 mL sterile water to be added to 200 mL of milk (3.75 mg/L and 5 mg/L, respectively). Subjects in the control group received tubes with a daily amount of 0.5 mL sterile water to be added to 200 mL of milk. The subjects were asked to consume the solution slowly from a glass during their evening meal. The fluoride solution was delivered from a pharmaceutical retailer (Apoteket AB, Sweden). Doses were filled into 1-mL tubes at the Department of Oral Biology, Malmö University, Sweden. The tubes were packed in marked boxes for each day, which the subjects were asked to store at room temperature. As illustrated in Figure 1, the subjects were provided with the tubes every second month at their public dental clinic. The natural fluoridation level of the water supply in the three communities was between 0.2 and 0.3 mg fluoride/L (personal communication from the responsible environmental inspector of each local community). No additional fluoride supplementation is made to any of the water supplies.

During the study, the subjects were asked to continue their usual oral hygiene regimes, including tooth-brushing twice a day with toothpaste containing fluorides (recommendation by the Swedish National Board of Health and Welfare). The subjects had visited their local Swedish Public Dental Health Service clinic annually since the age of 3, when they were instructed and shown digital resources on how to brush their teeth by a dental nurse or dental hygienist.

The subjects followed their ordinary oral care schedule at their local clinic, which coincided with the baseline and follow-up at 2 years. Data concerning health status, use of antibiotics, lactose intolerance, daily consumption of milk, tooth-brushing frequency, type of toothpaste, and use of other fluoride additives were collected. As was information on adverse effects (dental pain, visits to other dental clinics, antibiotics due to dental infection). Data were collected in individual logbooks designed for this study.

### Adherence

During the study, one appointed staff-member at each clinic was responsible for personal contacts with the subjects at their visits to the clinic. To support trust between the subject and the appointed staff-member, a dialogue was initiated and continued during the study time with each subject about adherence and how to incorporate advice and management of the solutions into their daily routine. At the visits to the clinic, the new, labelled boxes with the solutions were given to the subjects and the used boxes were returned to the staff-member, who at the same time handed over a chart with a 2-month schedule to be filled in daily at their evening meals. The chart was returned to the staff-member at the subjects’ next visit to the clinic.

### Caries registration

**Figure 2:**
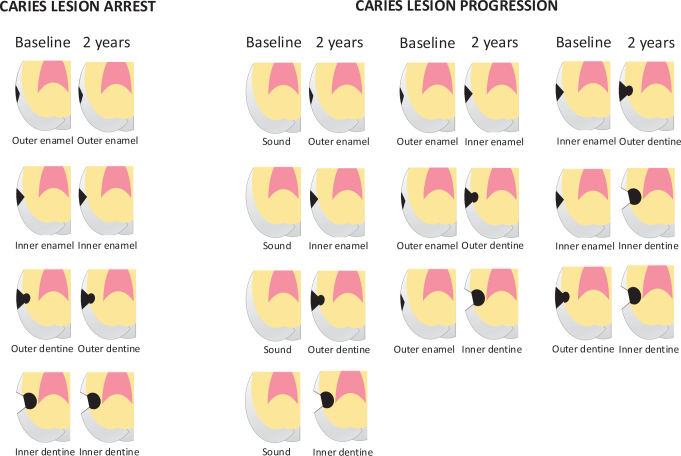
Radiological classification system of caries lesion arrest and caries lesion progression in proximal surfaces of premolars and molars. Adopted from Senneby et al. [21].

At baseline and 2 years, surfaces were assessed visually in line with ICDAS except for the proximal surfaces of the premolars and molars, which were assessed with a radiological classification system (Figures 1 and 2).

*Visual examination* was performed by three general dental practitioners (one at each clinic) who had more than 25 years of experience of examining adolescents’ caries status. Teeth were air-dried and surfaces examined under lighting with a mirror. Caries status was classified for the occlusal/incisal, buccal, and lingual surfaces of all teeth, and the proximal surfaces of incisors and cuspids in line with the ICDAS criteria: code 0 (sound), codes 1, 2, or 3 (enamel caries), and codes 4, 5, or 6 (dentine caries) [20].

*Radiographic examination* of the proximal surfaces of premolars and molars with one bitewing radiograph on each side of the mouth was performed with dental X-ray units (Planmeca Intra, Finland) operating at 8 mA and 63 kV with a rectangular positioning device and a focus-to-skin distance of 0.35 m. Two imaging plate systems used were Digora® (Soredex, Finland) with a 40 × 30 mm^2^ effective area or VistaScan® (Dürr Dental AG, Germany) with a 28 × 36 mm^2^ effective area. The imaging plates offered pixel sizes of 39 × 39 mm^2^ and 45 × 45 mm^2^, respectively. The imaging plates were enveloped in a plastic sheet and fixed to a Kwik-Bite® film holder (Kerr Corporation, US). The X-ray units were equipped with electronic timers and the exposure time was set to 0.25 s.

Prior to the baseline examination, a certified maxillofacial radiologist checked the dedicated radiographic equipment used throughout the whole study at each of the three clinics for the following parameters: tube voltage, exposure time (reproducibility and linearity), low contrast, and spatial resolution. The quality control of the X-ray units is previously presented in detail [21].

All images were exported as digital imaging and communication in medicine files to portable storage media from digital viewing software programmes. The images were then imported to Romexis® software (Planmeca, Finland) and anonymised. The computer and monitor used were visually adjusted with test images. The viewing room had dimmed lighting (ambient light below 20 lux) and the viewing distance to the screen was 30–40 cm. No image enhancement or altering of images was allowed, and bilinear interpolation for scaling of images on screen was used.

Baseline and follow-up bitewing images were displayed and assessed side-by-side by two raters together. Each proximal surface was assessed according to the radiological classification system of caries lesion development |^21^] as presented in Figure 2, which was available to the raters during the assessment. Caries enamel lesions were confined to the outer half of the enamel and lesions more than halfway through the enamel but not passing the enamel-junction junction. Caries lesions in dentine were confined to the outer half of the dentine and lesions more than halfway through the dentine.

### Training and calibration

Training and calibration of ICDAS criteria took place twice per year in two settings: (1) in each of the three practitioners’ clinics and (2) at a 2-day meeting for all participating staff-members from the dental clinics and the university.

The reason for asking two raters to assess the bitewing images together was to ensure that the images were displayed correctly. The two raters were well acquainted with the classification system (Figure 2). Their professional experience in maxillofacial radiology was 10 and 36 years, respectively. The intra-rater agreement for the raters together for assessment of lesion arrest and lesion progression was 96%, kappa being 0.63 (confidence interval (CI) 0.42–0.83).

### Sample size

When the project began, there was a lack of published data on caries increment including enamel and dentine in adolescents aged 12–19 years in scientific studies (search via the medical literature analysis and retrieval system online (MEDLINE)) or in the national statistical data (written data and personal communication with The Swedish National Board of Health and Welfare). Therefore, to determine sample size, we assumed a figure of 2% in the intervention group and 10% in the control group for new caries development. For an individually randomised study at a power of 80% with a significance level of 0.05, 140 subjects per group were required. To accommodate for a 20% drop-out rate, our goal was to invite 350 adolescents to the study.

### Randomisation and blinding

Randomisation was carried out by a senior researcher, who was not a co-author of the study. Each subject was allotted a unique code consisting of three random numbers, which were computer-generated for each clinic. After the randomisation, copies of the lists with code numbers were sent separately to each of the three clinics. The original lists were saved as sealed files in a safety-deposit box with the senior researcher. The tubes with fluoride and water solutions were distributed according to the randomisation list in tubes pre-labelled with the code numbers. The tubes had the same design independent of content of solution with fluoride or with only water (placebo). At the visual follow-up examination, the group assignment and data of previous examinations were not available to the general dental practitioners. Like the general dental practitioners, the subjects and their parents/guardians, the staff-members delivering the tubes, the trial investigators, and the outcome assessors and raters were blind to the group allocation. The code was broken only when the assessment of the outcomes had been processed.

### Outcome

Caries development in enamel and dentine after intervention with fluoridated milk over a 2-year period at three clinics.

### Primary outcome measures

- Caries lesion arrest: Lesions located at the occlusal/incisal, buccal, and lingual surfaces of all teeth, and the proximal surfaces of incisors and cuspids were assigned the same ICDAS code following a visual examination at both baseline and 2 years. The same coding was also used for proximal lesions in premolars and molars that were assessed radiologically showing no change over 2-year span (caries lesion arrest in Figure 2).
- Caries lesion progression: Lesions located at the occlusal/incisal, buccal, and lingual surfaces of all teeth, and the proximal surfaces of incisors and cuspids were assigned a higher ICDAS code following a visual examination at both baseline and 2 years. The same coding was also used for proximal lesions in premolars and molars that were assessed radiologically showing progression over 2 years (see Figure 2).

### Secondary outcome measures

- Caries DMFS increment for enamel lesions: Decayed, missing and filled surfaces including enamel lesions.
- Caries DMFS increment for dentine lesions: Decayed, missing and filled surfaces including dentine lesions.

### Assessment of outcomes

After the completion of the intervention at 2 years, the records of the visual examinations at baseline and follow-up were compared for occlusal/incisal, buccal, and lingual surfaces of all teeth, and the proximal surfaces of incisors and cuspids using ICDAS [20]. Each proximal surface of molars and premolars was assessed according to the radiological classification system of caries lesion development (Figure 2).

### Statistical methods

For all variables that were considered as numerical, two tests were used in the group comparisons (intervention/control): firstly, a *t*-test for two independent groups and secondly, the Mann-Whitney rank-sum test (*U*-test). In comparisons of the clinics, a one-way Analysis of Variance (ANOVA) or Tukey’s test were used. For all categorical variables, Chi-square tests and, in the case of small, expected frequencies, Fisher’s Exact test were used.

## Results

### Participant flow

The number of adolescents assessed as eligible was 292 and of these 87 in the intervention group and 72 in the control group returned every second month to their public dental clinic to collect their fluoride or water-containing tubes and for examination at 2 years (Figure 1). Subjects lost to follow-up comprised 44% of the intervention group and 47% of the control group. These subjects did not revisit the clinic at the scheduled time. The major reason for the loss was an anti-fluoride debate which influenced the subjects’ willingness to participate. Other reasons were life events, such as a move and thereby change of dental clinic, parents’ divorce, or sickness. In one of the three clinics, coronavirus disease 2019 (COVID-19) caused withdrawals of several adolescents.

## Recruitment

The periods of recruitment were different for the clinics: A—2012–2017, B—2013–2018, and C—2012–2013. When the clinical and university teams experienced that the recruitment was obstructed by the national and regional debate about assumed toxicity of fluorides, we decided to end recruitment of subjects although the inclusion rate was lower than planned.

## Baseline characteristics

Mean age was 12.6 years (range 11.7–14.1) and the percentage of male subjects ranged between 36% and 39% in intervention groups and between 40% and 53% in control groups. Dental status of the participating subjects at baseline is presented in Table 1. For all clinics together or for any single clinic, there was no statistical difference between the intervention and control groups regarding DMFS- or decayed, missing, filled teeth (DMFT)-values using *t*-test. At clinic A, the mean number of enamel lesions was higher in the intervention than in the control group (*t*-test, *p* = 0.041) including all surfaces.

**Table 1:** Dental status at baseline of subjects randomised to participate and followed for 2 years.

Dental status	All clinics together		Clinic A		Clinic B		Clinic C	
	Intervention (*n* = 87)	Control (*n* = 72)	Intervention (*n* = 44)	Control (*n* = 40)	Intervention (*n* = 25)	Control (*n* = 15)	Intervention (*n* = 18)	Control (*n* = 17)
**Caries-free (% subjects)**								
In enamel In dentine In enamel + dentine	60 86 58	69 89 60	66 84 59	75 80 58	52 84 52	60 100 60	61 94 61	65 100 65
**Mean DFS (SD) [Median DFS]**								
D including enamel and dentine lesions D including dentine lesions only	2.0 (2.9) [1] 1.1 (2.0) [0]	1.3 (1.8) [1] 0.8 (1.5) [0]	2.3 (3.3) [1] 1.5 (2.5) [0]	1.9 (1.9) [1] 1.0 (1.8) [0]	1.9 (2.6) [1] 0.7 (1.2) [0]	1.4 (1.9) [0] 0.3 (0.6) [0]	1.2 (2.2) [1] 0.6 (1.3) [0]	1.3 (1.4) [1] 0.7 (1.2) [0]
**Mean DFT (SD) [Median DFT]**								
D including enamel and dentine lesions D including dentine lesions only	1.7 (2.4) [1] 0.9 (1.5) [0]	1.2 (1.6) [1] 0.7 (1.3) [0]	1.8 (2.5) [1] 1.1 (1.8) [0]	1.3 (1.7) [1] 0.9 (1.6) [0]	1.9 (2.6) [1] 0.7 (1.2) [0]	1.3 (1.7) [0] 0.2 (0.4) [0]	1.1 (1.8) [1] 0.5 (1.2) [0]	1.1 (1.3) [1] 0.5 (0.8) [0]
**Mean number of caries lesions (SD)** **[Median number of caries lesions]**								
In enamel In dentine In occlusal and smooth surfaces In proximal surfaces	0.9 (1.4) [0] 0.2 (0.5) [0] 0.5 (1.2) [0] 0.6 (1.1) [0]	0.6 (1.0) [0] 0.2 (0.6) [0] 0.4 (0.8) [0] 0.4 (0.8) [0]	0.8 (1.2) [0] 0.2 (0.5) [0] 0.3 (0.7) [0] 0.6 (1.0) [0]	0.3 (0.7) [0] 0.4 (0.8) [0] 0.4 (0.9) [0] 0.3 (0.5) [0]	1.2 (2.0) [0] 0.2 (0.5) [0] 1.1 (1.8) [0] 0.4 (1.0) [0]	1.1 (1.7) [0] 0.0 (0.0) [0] 0.6 (1.1) [0] 0.5 (1.0) [0]	0.7 (1.1) [0] 0.1 (0.2) [0] 0.0 (0.0) [0] 0.7 (1.3) [0]	0.6 (0.9) [0] 0.0 (0.0) [0] 0.1 (0.2) [0] 0.5 (0.9) [0]
**Mean number of fillings (SD)** **[Median number of fillings]**								
Based on surfaces Based on teeth	0.9 (1.8) [0] 0.7 (1.3) [0]	0.6 (1.2) [0] 0.5 (0.9) [0]	1.3 (2.3) [0] 1.0 (1.6) [0]	0.7 (1.4) [0] 0.6 (1.1) [0]	0.5 (1.0) [0] 0.5 (1.0) [0]	0.3 (0.6) [0] 0.2 (0.4) [0]	0.5 (1.2) [0] 0.4 (1.0) [0]	0.7 (1.2) [0] 0.5 (0.8) [0]

*n*: number of subjects; DFS: decayed/filled surfaces; DFT: decayed/filled teeth; SD: standard deviation; *M*: missing was excluded in DMFS and DMFT as there were no missing teeth at baseline.

### Numbers analysed

For each group, the number of participants included in each analysis can be seen in tables.

### Caries DFS (decayed/filled surfaces) increment

No teeth were lost in any subject over the 2-year period. Therefore, M was excluded from DMFS in the following text and Table 2. Decayed/filled surfaces increment including both enamel and dentine lesions for each subject at 2 years is presented in Supplementary figure. In the intervention groups, 70% of the subjects at clinic A, 64% at clinic B, and 94% at clinic C displayed unchanged decayed, filled surface (DFS)-values. Corresponding proportions in the control groups were 30%, 36%, and 6%, respectively. Notably, only one subject in the intervention group at clinic C had new caries lesions.

**Table 2:** Decayed/Filled Surfaces increment and incidence in intervention and control group at 2-year follow-up.

Clinic	Group	Mean DFS increment (SD) including			Incidence of DFS increment (%) including		
		Enamel lesions	Dentine lesions	All lesions	Enamel lesions	Dentine lesions	All lesions
**All clinics**	**Intervention (*n* = 87)**	0.4 (1.3)	0.3 (0.7)	0.7 (1.5)	21	18	26
**Together**	**Control (*n* = 72)**	0.8 (2.0)	0.5 (1.0)	1.3 (2.3)	38	26	46
	*P*	*0.08^T^*	*0.23^T^*	*0.04^T^*	*0.02^C^*	*0.23^C^*	*0.01^C^*
**Clinic A**	**Intervention (*n* = 44)**	0.4 (1.4)	0.4 (1.0)	0.8 (1.5)	20	20	30
	**Control (*n* = 40)**	0.9 (2.3)	0.5 (1.0)	1.4 (2.6)	33	30	43
	*P*	*0.22^T^*	*0.67^T^*	*0.20^T^*	*0.21^C^*	*0.31^C^*	*0.22^C^*
**Clinic B**	**Intervention (*n* = 25)**	0.7 (1.6)	0.2 (0.4)	0.9 (1.7)	32	16	36
	**Control (*n* = 15)**	1.2 (2.0)	0.7 (1.4)	1.9 (2.2)	53	27	67
	*P*	*0.40^T^*	*0.20^T^*	*0.13^T^*	*0.18^C^*	*0.44^F^*	*0.06^C^*
**Clinic C**	**Intervention (*n* = 18)**	-0.1 (0.5)	0.2 (0.4)	0.1 (0.2)	6	17	6
	**Control (*n* = 17)**	0.4 (0.6)	0.2 (0.4)	0.6 (0.9)	35	18	35
	*P*	*0.01^T^*	*0.94^T^*	*0.04^T^*	*0.04^F^*	*1.00^F^*	*0.04^F^*
Clinic	Group	Reduction of mean DFS increment including			Reduction of incidence of DFS increment (%) including		
		Enamel lesions	Dentine lesions	All lesions	Enamel lesions	Dentine lesions	All lesions
**All clinics**	**Difference**	0.5	0.2	0.6	16.8	8.0	19.4
**together**	95% CI for difference	*-0.1 – 1.0*	*-0.1 – 0.5*	*0.02 – 1.3*	*2.8 – 30.8*	*-5.1 – 21.1*	*4.6 – 34.2*
**Clinic A**	**Difference**	0.5	0.1	0.6	12.0	9.5	13.0
	95% CI for difference	*-0.3 –1.3*	*-0.3 – 0.5*	*-0.3 – 1.5*	*-7.1 – 31.2*	*-9.4 – 28.5*	*-7.9 – 33.8*
**Clinic B**	**Difference**	0.5	0.5	1.0	21.3	10.7	30.7
	95% CI for difference	*-0.7 – 1.6*	*-0.3 – 1.3*	*-0.3 – 2.3*	*-11.2 – 53.8*	*-16.2 – 37.6*	*-1.7 – 56.1*
**Clinic C**	**Difference**	0.5	0.0	0.5	29.7	1.0	29.7
	95% CI for difference	*0.1 – 0.9*	*-0.3 – 0.3*	*0.0 – 1.0*	*3.4 –56.1*	*-25.7 – 27.7*	*3.4 – 56.1*

Differences calculated using *t*-test (*T*), Chi-2 test (*C*) or Fisher’s Exact test (*F*). n: number of subjects; DFS: decayed/filled surfaces; SD: standard deviation; CI: confidence of interval; M: missing was excluded in DMFS as there were no missing teeth at 2-year follow-up.

For all clinics together, the mean number of surfaces with enamel or dentine caries was significantly lower in the intervention group than in the control group (Table 2). In addition, the incidence of new enamel lesions was lower in the intervention group indicating that fluoride primarily affected development of enamel caries. Clinic C contributed the most to these differences. There was no difference between the arms for reduction of mean DFS increment or incidence including only dentine lesions (Table 2).

### Caries lesion arrest

For all clinics together, the mean number of caries lesions arrested in the enamel of all surfaces and in the outer part of the enamel at proximal surfaces was significantly higher in the intervention group (Table 3). There was no difference between the intervention and control groups for the outer dentine. Caries lesion arrest was not observed in the inner dentine in any group or clinic. At clinic A, the mean number of surfaces with caries lesion arrest including only enamel or including both enamel and dentine was significantly higher in the intervention than in the control group when including all surfaces (occlusal, smooth, and proximal) or proximal surfaces only (Table 3). The mean number of lesion arrests in the outer enamel of proximal surfaces was significantly higher in the intervention group at clinic A (Table 3).

**Table 3:** Caries lesion arrest in intervention and control group at 2-year follow-up.

Clinic	Group	Mean number of all surfaces (SD) with caries lesion arrest						Incidence of caries lesion arrest (%)					
		In enamel		In dentine		Enamel + Dentine		In enamel		In dentine		Enamel + Dentine	
		All	Proximal	All	Proximal	All	Proximal	All	Proximal	All	Proximal	All	Proximal
**All clinics**	**Intervention (*n* = 87)**	0.7 (1.3)	0.3 (0.7)	0.1 (0.4)	0.1 (0.3)	0.8 (1.5)	0.4 (0.9)	32	22	7	6	33	23
**together**	**Control (*n* = 72)**	0.3 (0.7)	0.2 (0.4)	0.0 (0.1)	0.0 (0.1)	0.3 (0.7)	0.2 (0.4)	24	17	1	1	25	18
	*P*	*0.04^T^*	*0.06^T^*	*0.06^T^*	*0.12^T^*	*0.02^T^*	*0.04^T^*	*0.23 ^C^*	*0.41 ^C^*	*0.13 ^F^*	*0.22^F^*	*0.25 ^C^*	*0.45 ^C^*
													
**Clinic A**	**Intervention (*n* = 44)**	0.5 (0.9)	0.3 (0.7)	0.1 (0.3)	0.0 (0.2)	0.5 (1.0)	0.4 (0.8)	27	25	5	2	30	25
	**Control (*n* = 40)**	0.2 (0.4)	0.1 (0.3)	0.0 (0.2)	0.0 (0.2)	0.2 (0.4)	0.1 (0.3)	13	8	3	3	15	10
	*P*	*0.04^T^*	*0.02^T^*	*0.46^T^*	*0.95^T^*	*0.049^T^*	*0.04^T^*	*0.09^C^*	*0.03 ^C^*	*1.00 ^F^*	*1.00 ^F^*	*0.11 ^C^*	*0.07 ^C^*
													
**Clinic B**	**Intervention (*n* = 25)**	1.2 (2.0)	0.2 (0.6)	0.2 (0.5)	0.2 (0.5)	1.4 (2.2)	0.4 (1.0)	44	12	12	12	44	16
	**Control (*n* = 15)**	0.8 (1.1)	0.3 (0.5)	0.0 (0.0)	0.0 (0.0)	0.8 (1.1)	0.3 (0.5)	40	27	0	0	40	27
	*P*	*0.48^T^*	*0.71^T^*	*0.10^T^*	*0.10^T^*	*0.37^T^*	*0.72^T^*	*0.80^C^*	*0.39 ^F^*	*0.28 ^F^*	*0.28 ^F^*	*0.80^C^*	*0.44 ^F^*
													
**Clinic C**	**Intervention (*n* = 18)**	0.5 (0.9)	0.5 (0.9)	0.1 (0.2)	0.1 (0.2)	0.6 (1.1)	0.6 (1.1)	28	28	6	6	28	28
	**Control (*n* = 17)**	0.4 (0.5)	0.3 (0.5)	0.0 (0.0)	0.0 (0.0)	0.4 (0.5)	0.3 (0.5)	35	29	0	0	35	29
	*P*	*0.56^T^*	*0.41^T^*	*0.33^T^*	*0.33^T^*	*0.49^T^*	*0.36^T^*	*0.63^C^*	*1.00 ^F^*	*1.00 ^F^*	*1.00 ^F^*	*0.63^C^*	*1.00 ^F^*
Clinic	Group	Mean number of proximal surfaces (SD) with caries lesion arrest						Incidence of proximal caries lesion arrest (%)					
		In outer enamel		In inner enamel		In outer dentine		In outer enamel		In inner enamel		In outer dentine	
**All clinics**	**Intervention (*n* = 87)**	0.3 (0.7)		0.0 (0.2)		0.1 (0.3)		18		3		6	
**together**	**Control (*n* = 72)**	0.1 (0.3)		0.0 (0.2)		0.0 (0.1)		13		4		1	
	*P*	*0.04^T^*		*0.81^T^*		*0.12^T^*		*0.31^C^*		*1.00 ^F^*		*0.22 ^F^*	
							
**Clinic A**	**Intervention (*n* = 44)**	0.3 (0.7)		0.0 (0.2)		0.0 (0.2)		20		5		2	
	**Control (*n* = 40)**	0.1 (0.3)		0.0 (0.0)		0.0 (0.2)		8		0		3	
	*P*	*0.048^T^*		*0.16^T^*		*0.95^T^*		*0.09 ^C^*		*0.50 ^F^*		*1.00 ^F^*	
							
**Clinic B**	**Intervention (*n* = 25)**	0.2 (0.6)		0.0 (0.0)		0.2 (0.5)		12		0		12	
	**Control (*n* = 15)**	0.2 (0.4)		0.1 (0.3)		0.0 (0.0)		20		7		0	
	*P*	*1.00^T^*		*0.33^T^*		*0.10^T^*		*0.65 ^F^*		*0.38 ^F^*		*0.28 ^F^*	
							
**Clinic C**	**Intervention (*n* = 18)**	0.4 (0.9)		0.1 (0.2)		0.1 (0.2)		22		6		6	
	**Control (*n* = 17)**	0.2 (0.4)		0.1 (0.3)		0.0 (0.0)		18		12		0	
	*P*	*0.27^T^*		*0.53^T^*		*0.33^T^*		*1.00 ^F^*		*0.60 ^F^*		*1.00 ^F^*	

*n*: number of subjects; SD: standard deviation. Differences calculated using *t*-test (*T*), Chi-2 test (*C*) or Fisher’s Exact test (*F*).

**Figure 3:**
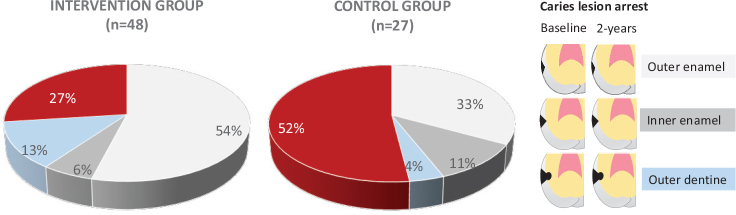
Proportion of caries lesion arrest at proximal surfaces of premolars and molars in intervention and control group for all clinics together. The classification system for radiological assessment is presented lateral to the pie chart. The total number of surfaces with lesions at baseline was 48 in intervention group and 27 in control group. At 2-year follow-up the proportion of caries lesion arrest was 73% in the intervention group and 48% in the control group. The proportion of caries lesion progression is displayed in red. In the intervention group, 27% of all lesions at baseline showed progression while the corresponding figure for the control group was 52%.

To further analyse the effect of the intervention, all proximal lesions in premolars and molars at all clinics together at baseline were taken into consideration and compared to lesion arrest data at 2 years (Figure 3). Despite the skewed distribution of numbers of proximal caries lesions at baseline, it was possible to demonstrate differences in lesion arrest between the groups. The proportion of lesion arrest was higher in the intervention group (73%) than in the control group (48%) (*p* = 0.05; Fisher’s Exact test). Furthermore, the proportion of caries lesion arrest in the outer half of the enamel was higher in the intervention group than in the control group (*p* = 0.06; Fisher’s Exact test) suggesting that fluoride mainly exerts its effect on lesions in the outer half of the enamel.

### Caries lesion progression

At all clinics together, the mean number of caries lesions progressing to or within enamel together with to or within dentine was lower in the intervention group than in the control group (Table 4). At clinic C, the mean number of all surfaces with progression to or within enamel was lower after intervention, while there was no difference between the groups at clinics A and B (Table 4). The incidence of caries lesion progression to or within enamel at all clinics together was statistically lower in the intervention group compared to the control group. These outcomes again indicate that fluoride primarily influenced caries progression in enamel.

**Table 4:** Caries lesion progression in intervention and control group at 2-year follow-up.

Clinic	Group	Mean number of all surfaces (SD) with caries lesion progression			Incidence of caries lesion progression (%)		
		To or within enamel	To or within dentine	To or within enamel + to or within dentine	To or within enamel	To or within dentine	To or within enamel + to or within dentine
**All clinics**	**Intervention (*n* = 87)**	0.6 (1.3)	0.4 (0.9)	0.9 (1.6)	25	21	37
**together**	**Control (*n* = 72)**	1.1 (2.1)	0.6 (1.1)	1.7 (2.6)	42	32	56
	*P*	*0.08^T^*	*0.14^T^*	*0.04^T^*	*0.03^C^*	*0.11^C^*	*0.02^C^*
**Clinic A**	**Intervention (*n* = 44)**	0.6 (1.4)	0.5 (1.2)	1.2 (1.8)	30	25	41
	**Control (*n* = 40)**	1.1 (2.3)	0.8 (1.3)	1.9 (2.9)	38	40	58
	*P*	*0.32^T^*	*0.31^T^*	*0.19^T^*	*0.44^C^*	*0.14^C^*	*0.13^C^*
**Clinic B**	**Intervention (*n* = 25)**	0.8 (1.6)	0.2 (0.5)	1.0 (1.7)	32	16	40
	**Control (*n* = 15)**	1.5 (2.4)	0.5 (1.1)	2.1 (2.8)	53	27	67
	*P*	*0.22^T^*	*0.27^T^*	*0.13^T^*	*0.18^C^*	*0.44 ^F^*	*0.10 ^C^*
**Clinic C**	**Intervention (*n* = 18)**	0.1 (0.2)	0.2 (0.4)	0.2 (0.4)	6	17	22
	**Control (*n* = 17)**	0.7(0.9)	0.2 (0.4)	0.8 (1.3)	41	18	41
	*P*	*0.02^T^*	*0.94^T^*	*0.08^T^*	*0.02 ^F^*	*1.00 ^F^*	*0.23^C^*
Clinic	Group	Mean number of proximal surfaces (SD) with caries lesion progression			Incidence of proximal caries lesion progression (%)		
		To or within enamel	To or within dentine	To or within enamel + to or within dentine	To or within enamel	To or within dentine	To or within enamel + to or within dentine
**All clinics**	**Intervention (*n* = 87)**	0.5 (1.2)	0.1 (0.5)	0.6 (1.3)	21	12	28
**together**	**Control (*n* = 72)**	0.7 (1.2)	0.2 (0.5)	0.8 (1.4)	35	10	39
	*P*	*0.22*	*0.97*	*0.27*	*0.05^C^*	*0.72^C^*	*0.13^C^*
**Clinic A**	**Intervention (*n* = 44)**	0.6 (1.2)	0.2 (0.6)	0.7 (1.3)	27	11	34
	**Control (*n* = 40)**	0.6 (1.2)	0.1 (0.4)	0.7 (1.3)	28	10	33
	*P*	*0.84*	*0.61*	*0.94*	*0.98^C^*	*1.00^F^*	*0.88^C^*
**Clinic B**	**Intervention (*n* = 25)**	0.6 (1.6)	0.1 (0.3)	0.7 (1.7)	20	8	20
	**Control (*n* = 15)**	1.1 (1.4)	0.3 (0.9)	1.4 (1.8)	47	13	53
	*P*	*0.35*	*0.31*	*0.22*	*0.09^F^*	*0.62^F^*	*0.04^F^*
**Clinic C**	**Intervention (*n* = 18)**	0.1 (0.2)	0.2 (0.4)	0.2 (0.4)	6	17	22
	**Control (*n* = 17)**	0.6 (0.9)	0.1 (0.2)	0.6 (1.0)	41	6	41
	*P*	*0.03*	*0.33*	*0.12*	*0.02^F^*	*0.60^F^*	*0.23^C^*

SD: standard deviation. Differences calculated using *t*-test (*T*-test), Chi-2 test (*C*) or Fisher’s exact test (*F*).

For all clinics together, there was no significant difference between the total mean number of proximal surfaces with caries progression (Table 4). Mean number of proximal surfaces with caries lesion progression to or within enamel was lower in the intervention group at clinic C (Table 4). The incidence of caries lesion progression to or within in enamel for proximal surfaces at clinic C and all clinics together was statistically lower in the intervention compared to the control group.

**Figure 4:**
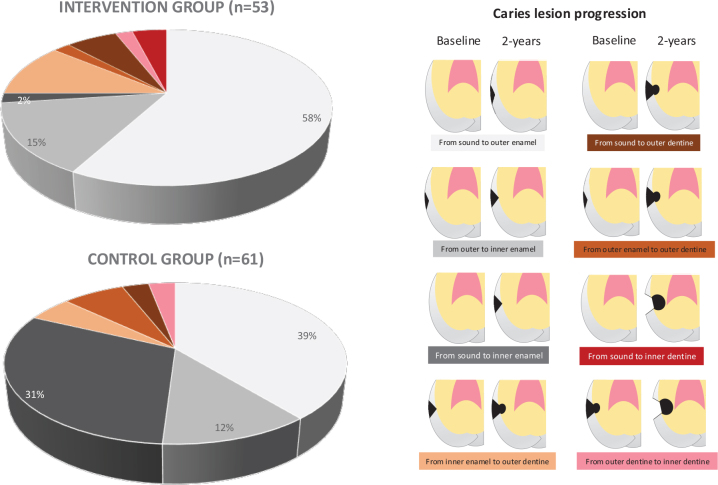
Proportion of different types of caries lesion progression at proximal surfaces of premolars and molars in intervention group and control group for all clinics together. The classification system for radiological assessment is presented lateral to the pie chart.

Figure 4 presents proportions of caries lesion progression at proximal surfaces of premolars and molars at all clinics together. At 2 years, the total number of lesions that progressed in the intervention and control groups was of the same magnitude. However, the comparison of the baseline and 2-year follow-up images visualised different types of lesion progression, which is presented in Figure 4. The lower proportion of lesions proceeding from sound to inner half of enamel over 2 years was significantly lower in the intervention group (2% of lesions) than in the control group (31% of lesions) (*p* = 0.00; Fisher’s Exact test). However, there was a tendency towards a higher proportion of lesions progressing from sound to the outer half of enamel in the intervention group (58%) than in the control group (39%). We interpret these findings as slower rate of progression (from sound to outer half of enamel) in the intervention group compared to the control group, where progression was more rapid (from sound to inner half of enamel).

### Harms and adverse effect

The daily doses of fluoride were below those recommended for prophylactic use in caries prevention in adolescents. At 12–13 years of age permanent teeth, except for the third molars, are developed and the risk for dental fluorosis was therefore not considered. There was no report of adverse effects listed in methods (dental pain, visits to other dental clinics, antibiotics due to dental infection).

## Discussion

### Key findings and implications for caries prevention

Low doses of fluoride administered daily in milk, in addition to oral hygiene routines with fluoridated toothpaste, reduced caries lesion progression, and increased caries lesion arrest. Furthermore, the percentage of individuals (incidence) of the intervention group with lesion progression to or within enamel at proximal surfaces was significantly lower than in the control group. These findings provide evidence for a preventative effect of fluoridated milk.

The proportion of lesion progression from sound to the inner enamel was significantly lower in the intervention group compared to the control group, while the proportion of lesion progression from sound to the outer half of enamel was higher. When it comes to arrested caries lesions, the proportion in the outer half of enamel of proximal surfaces was higher in the intervention group than in the control group. Based on these findings, we suggest that fluoride was adsorbed and accumulated at the surface of partially demineralised hydroxyapatite crystals which would decrease further demineralisation of enamel during pH falls generated by dental biofilm bacteria.

In adolescents, caries development at proximal surfaces is of special interest [2]. Considering that the greatest effect of fluoride was on lesions in the outer half of enamel of proximal surfaces, additional fluoride in milk could be given to individuals with these enamel caries lesions. This favours the notion of a tailored individualised approach based upon disease level and distribution to prevent and control enamel caries. This is a shift from universal prevention with fluorides towards targeted prevention. Such an approach must be underpinned by an early detection of enamel caries lesions.

### Methodological considerations

Twenty years ago, it was emphasised at an international consensus conference that the evaluation of anti-caries interventions should include the impact on non-cavitated lesions [22]. Following the philosophy of interceptive prevention, the efficacy of preventive agents on initial lesions is the key. Nevertheless, results of caries studies traditionally rely on examinations of cavitated lesions, which will disregard the continuum of the caries process over time [2, 22–24]. In line with the recommendations in the consensus report [22], we reported the reduction of DFS increment as one outcome measure. We consider, however, this measure to be inadequate to reflect preventive efficacy. Decayed/filled surfaces increment will for example be numerically identical irrespective whether a lesion in the outer part of the enamel progresses into the inner part of the enamel. With the radiological classification system applied in this study, it was possible to assess lesion development at proximal surfaces as lesion progression from sound to the outer part of enamel, or from the outer part to the inner part of enamel or to dentine. Assessment of minute changes over time requires images of high quality, which can be affected at any point along the imaging chain. Firstly, periodic-identical images regarding geometrical relationships are essential. Secondly, calibration of the X-ray units used at baseline and follow-up within a study is imperative to guarantee images of comparable density and resolution. Thirdly, the baseline and follow-up images were paired and assessed simultaneously according to the classification system [21]. The approach of the applied system is based on the fact when assessing signs of minute tissue changes (lesion progression) or no changes (lesion arrest) over time, the comparison of two images side by side involves a different mindset physiologically and psychologically than the assessment of two images viewed separately. The classification system was designed with side-by side illustrations – visual patterns – drawn to resemble proximal surfaces with and without lesion changes. This is in line with studies in visual diagnoses that when data are elicited as discrete features, there is a large perceptual component that rapidly recognises patterns (the concept of pattern recognition) rather than a cognitive component that seeks for further discrimination. Seeing features within an appropriate context induces familiarity effects and will underpin categorisation [25, 26].

### Limitations and strengths

The sample is limited but can provide important information that contributes to the larger body of evidence and be combined in meta-analyses [27]. We followed the notion by Guyatt et al. [27] ‘But is it not ethical to contribute to a body of knowledge that ultimately leads to a definite answer?’ We were aware prior to study that adolescents often experience an intense period of turmoil in their lives. This could result in losing interest in following routines. However, a limiting factor, which we did not foresee, was the intense, public anti-fluoride debate. This influenced not only recruitment but also the drop-out rate in the study.

We consider the high standard of the examinations by which the current study was conducted as a strength and that the findings of caries lesion development are both robust and unique. The radiological classification system was a prerequisite for the identification of various types of caries development. To our knowledge, the present study is one of few clinical longitudinal caries trials that have utilised a radiological classification system based on knowledge about how we see and perceive images [21]. As outcomes were based on two examinations, the baseline and the follow-up examinations with a long so called wash-out period of 2 years, intra-rater agreement of the visual examination expressed as kappa was unjustified. Reliability was ensured through repeated training and calibration.

The subjects have been informed annually about eating habits and instructed how to brush their teeth since they were aged 3. At the baseline examination and continuously during the study, the participants were asked about their oral hygiene and eating habits. There were no deviant records on toothbrushing or sugar consumption in their individual logbooks, containing data from the baseline examination as well as the visits every second month. As the present study used a randomised study design which ensures that each subject has an equal chance of being assigned to any group, we assumed that the intervention and control groups were comparable concerning toothbrushing and sugar consumption. Nevertheless, it is a challenge to identify outliers concerning tooth brushing and eating habits. As these factors may influence our results it should be considered as a possible limitation of the study.

A strength was that the subjects were seen at their ordinary clinics, where instructions about oral hygiene are designed and performed to give patients equivalent care. The participants were not selected patients visiting a university clinic. Thereby, the selection bias at the trial entry was reduced. Information about the total number of adolescents being asked to participate was not collected nor was the reason for not participating recorded. This may have affected the selection bias.

## Conclusions

Daily intake of fluoridated milk decreased DFS increment and progression of enamel caries, and increased caries arrest over a 2-year period. For the first time a radiological classification system with illustrations of caries lesion development over time was applied to discriminate between different kinds of caries progression. The results revealed that the proportion of lesion progressing from sound to inner enamel was significantly lower in the intervention group than in the control group. At the same time, there was a clear tendency towards a higher proportion of lesions progression from sound to outer enamel in the intervention group. We interpret these findings as slower progression (sound to outer enamel) in the intervention group compared to the control group where progression was more rapid (sound to inner enamel). Furthermore, the proportion of caries lesion arrests in the outer enamel was higher in the intervention group than in the control group. In all, we suggest that fluoride mainly exerts its effect on lesions in the outer enamel.

Generalisability defined as the applicability of the trial findings is limited. However, the transportability of the findings, that is, the applicability to another similar target population is relevant. Thus, based on the prevalence and skewed distribution of enamel caries in early adolescents, we propose that prevention should be targeted to those with enamel caries. Such a strategy, which can assist clinicians, public health leaders, and researchers in designing programmes for caries prevention in adolescents, should favour a shift from universal towards individualised prevention to control enamel caries lesions. Fluoridated milk represents one alternative to promote individual prevention.

## Data Availability

The datasets generated and analysed during the current study are available from the corresponding author on reasonable request.
